# Evaluation of Clinical and Radiological Outcomes of Anterior Cervical Hybrid Surgery for Multilevel Cervical Disc Disease

**DOI:** 10.7759/cureus.114146

**Published:** 2026-08-07

**Authors:** Bharatkumar R Dave, Arjit Vashishtha, Ajay Krishnan, Shivanand C Mayi, Ravi Ranjan Rai, Mirant B Dave, Mikeson Panthackel, Saurabh S Kulkarni, Yogenkumar Adodariya, Kishan Panjwani, Mukesh Patel

**Affiliations:** 1 Spine Surgery, Stavya Spine Hospital and Research Institute, Ahmedabad, IND; 2 Spine Surgery, BIMS, Bhavnagar Institute Of Medical Science, Bhavnagar, IND; 3 Orthopedics, Mahatma Gandhi Medical College and Research Institute, Aurangabad, IND; 4 Neurosurgery, Stavya Spine Hospital and Research Institute, Ahmedabad, IND

**Keywords:** anterior cervical discectomy and fusion (acdf), anterior cervical surgery, cervical tdr, hybrid cervical surgery, multilevel cervical disc disease

## Abstract

Introduction

While dealing with multilevel cervical disc disease, there is no consensus regarding the ideal treatment. Traditionally, anterior cervical discectomy and fusion (ACDF) has been the time-tested procedure with good outcomes for single-level pathologies, but it is associated with disadvantages, including disruption of the biomechanical environment, increased stresses on adjacent superior and inferior segments during movement, accelerated degeneration, and Adjacent Segment Disease (ASD). Cervical total disc replacement (TDR) has been proposed to prevent this by preserving the range of motion (ROM) at the level of pathology and reducing stresses on adjacent segments. However, as is the case with ACDF, the results of cervical TDR in multilevel cervical disc disease are inconsistent. As different levels are at different degrees of degeneration, hybrid surgery (HS) has been proposed as a combination of ACDF and TDR, which can theoretically preserve cervical spine movements and reduce abnormal loading at adjacent levels, thus preventing the ASD. However, clinical benefits are yet to be proven.

Materials and methods

We did a retrospective study of 20 patients who underwent HS at Stavya Spine Hospital and Research Institute. All patients were evaluated clinically and radiologically periodically for a minimum follow-up of two years. Demographic data and intra-operative findings and complications, if any, were noted from the patients’ records. Clinical evaluation included Visual Analog Scale (VAS) score, Neck Disability Index (NDI) score, and modified Japanese Orthopaedics Association (mJOA) score. Radiologically, cervical spine lateral view radiographs were done to assess C2-C7 ROM, adjacent superior and inferior segment ROM, and any evidence of heterotrophic ossification (HO).

Results

Of the 20 patients included, the mean age was 41.3 ± 7.4 years (range 36-62 years). The mean operative time was 125.8 ± 20.5 minutes, with a mean intraoperative blood loss of 138 ± 62.6 mL. The average follow-up period was 4.6 ± 3.7 years (ranging from 12 to 28 months). All the patients had statistically significant clinical improvement in terms of VAS score, NDI score, and mJOA score. Radiologically, mean C2-C7 ROM was 38.2° ± 9.6°, mean ROM at the superior adjacent segment was 8.2° ± 3.9°, and the inferior adjacent segment was 9.6° ± 4.3°. None of the patients exhibited any evidence of HO.

Conclusion

HS for multilevel disc disease in the cervical spine is associated with good clinical outcomes in terms of VAS, NDI, and mJOA scores. Radiologically, the benefit of HS is highlighted in terms of preserving the movements at the level of pathology, which reduces abnormal adjacent segment loading and thus potentially reduces the incidence of ASD.

## Introduction

The gold standard for the surgical management of cervical degenerative disc disease has long been anterior cervical discectomy and fusion (ACDF). The procedure has been time-tested for many years and is associated with patient satisfaction rates as high as 87% [1-4]. However, the fusion at the pathological level disturbs the biomechanics of the cervical spine and is associated with increased stress at the adjacent segments. Biomechanical studies have shown an increase in intra-discal pressure at the adjacent segments during motion, as high as 73% [5]. Hilibrand et al. [6] reported the occurrence of adjacent segment diseases (ASD) following ACDF at the rate of 2.9% per year for 10 years. Ishihara et al. [7] reported ASD following ACDF as 19% at two-year follow-up.

Since the advent of cervical total disc replacement (TDR), preservation of mobility at the pathological level and reduction in stress at the adjacent segments have been the ideology [8]. Over the years, evolving through various designs and technological advancements, the procedure has now been established as a safe and efficacious surgical option, with a reduction in the incidence of adjacent segment degeneration [9]. Zhong et al. reported lower re-operation rates for TDR (6%) as compared to ACDF (12%), which was statistically significant, in their meta-analysis of over 3,200 patients and 12 randomised controlled trials [10]. However, the procedure is contraindicated in severe spondylosis, ossification of the posterior longitudinal ligament (OPLL), instability, and facet arthritis.

For the surgical management of multilevel cervical disc diseases, many procedures have been described from an anterior or posterior approach, ranging from posterior laminectomies and lamino-foraminotomies to multi-level ACDFs or corpectomies. However, multi-level ACDFs have been shown to increase the shear strain at adjacent segments [11,12]. As different levels would be at different degrees of degeneration in multilevel cervical disc disease, all the pathological levels may not be amenable to management by TDR, as it is contraindicated in conditions such as facet arthritis, kyphosis, or instability. Multi-level TDRs have also been thought to alter the biomechanics of the cervical spine, possibly due to the artificial hypermobility [13].

The prospective study of Philips et al. has shown that the early clinical outcomes of TDR adjacent to an earlier fusion are comparable to the outcomes of primary TDR [14]. Following this, Lee et al. considered TDR adjacent to the fusion level for multi-level cervical disc disease as an attractive option to avoid multilevel fusion [15]. They demonstrated the biomechanical advantages of this hybrid (ACDF with TDR) construct in cadaveric models, including reduced adjacent segment stress. The early results of hybrid anterior cervical surgery done for multi-level cervical disc degeneration disease were published by Barbagallo et al. [16]. They concluded that the strategy is safe and efficacious. Many studies have been done to evaluate the role of the hybrid procedure for multi-level cervical disc degeneration [17-21].

The purpose of this study was to describe the clinical outcomes in terms of pain, disability, and neurological status and radiological outcomes in terms of range of motion (ROM) and implant status of the 20 patients who underwent hybrid surgery at our centre with a minimum follow-up period of two years.

This study was previously presented at the Bombay Spine Society annual conference (BSSCON) on 12 July 2025.

## Materials and methods

This is a retrospective study for the evaluation of clinical and radiological outcomes of patients who underwent hybrid surgery for multiple-level cervical spondylosis, with a minimum follow-up period of two years.

The protocol has been approved by the Institutional Ethics Committee (IEC approval number - SSHRI/CS/NS/Hybrid Cx/BRD/81/12.25, dated 4 December 2025) and registered with Clinical Trials Registry - India (CTRI/2025/12/098897).

Data collection

Out of 2,710 cervical spine surgeries done at Stavya Spine Hospital and Research Institute, Ahmedabad, Gujarat, India, we retrospectively evaluated 20 cases that underwent hybrid surgery for multilevel cervical disc disease, having a minimum follow-up period of two years after the surgery.

The primary inclusion criteria were multilevel cervical disc disease managed with hybrid surgery (at least 1 level ACDF and 1 level TDR), with hospital records of at least two years of follow-up after the surgery. The patients lost to follow-up or having inadequate records were excluded.

Demographic details and clinical data were obtained from the hospital records, and radiological evaluation was done based on the lateral radiographs of the cervical spine in flexion and extension positions.

Clinical evaluation was done in terms of the Visual Analog Scale (VAS) [22], Neck Disability Index (NDI) [23], and the modified Japanese Orthopaedics Association (mJOA) score [24]. The rationale behind choosing VAS was to have an objective assessment of pain. NDI provides a parameter to measure the disability in daily activities due to the pain, while the mJOA score reflects the disability due to the myelopathy in patients included in the study.

Radiological assessment was done based on the lateral cervical radiographs in the flexion and extension positions of the neck. The parameters measured were the C2-C7 ROM, the superior and inferior adjacent segments' ROM, and heterotopic ossification, if any.

Data analysis

All the obtained demographic, clinical, and radiological data from the time of presentation and intra-operative details, including the duration of surgery and peri-operative blood loss, as well as clinical and radiological data from the follow-up visits were recorded in tabular form and analysed in Microsoft Excel (Microsoft Corporation, Redmond, Washington). Demographic and quantitative variables are described as mean ± standard deviation and range (minimum-maximum).

Surgical technique

Under general anaesthesia, in the supine position, after confirmation of the pathological levels with the C-arm, exposure is done with Smith-Robinson's approach. The pathological level more severely affected is chosen for ACDF, while the level with a lesser degree of affection, without facet arthropathy or calcification in the disc, is chosen for TDR. If both the levels are equally degenerated, the level with more inherent mobility is chosen for TDR (C5-C6 having more inherent mobility than C6-7). Mobility of the TDR prosthesis is assessed by flexion and extension with the C-arm. The wound is closed in layers with a drain. The patient is encouraged to mobilise on the second day of surgery.

## Results

Of the 20 patients included in the study, the mean age was 41.3 ± 7.4 years. There were 12 males and eight female patients. The mean follow-up period was 4.6 ± 3.7 years following surgery. The demographic data are represented in Table 1.

**Table 1:** Demographic characteristics of the study cohort. The table summarises the baseline demographic data of the 20 patients included in the study, including age, sex distribution, and duration of follow‑up after surgery.

Variable	Value
Total number of patients	20
Mean age (years)	41.3 ± 7.4
Male (n, %)	12 (60%)
Female (n, %)	8 (40%)
Mean follow-up after surgery (years)	4.6 ± 3.7

Seventeen out of 20 patients underwent double-level surgery, whereas three underwent surgery at three levels. Three out of 20 patients were operated on non-contagious levels. Eight patients had ACDF done cranial to the TDR, while in 12 patients, ACDF was done at the level caudal to the TDR. The most commonly performed combination was C4-C5 TDR with C5-C6 ACDF (n = 6/20), followed by C5-C6 ACDF with C6-C7 TDR (n = 5/20). The mean duration of surgery was found to be 125.8 ± 20.5 minutes, whereas the mean intra-operative blood loss was found to be 138 ± 62.6 mL. The surgical characteristics are summarised in Table 2.

**Table 2:** Surgical characteristics of the study cohort. The table summarises the extent of surgery (double‑ vs three‑level), proportion of non‑contiguous levels, the location of anterior cervical discectomy and fusion (ACDF) relative to total disc replacement (TDR), and the two most frequently used surgical level combinations, along with mean duration of surgery (minutes) and mean intra-operative blood loss, in the 20‑patient cohort. ACDF: anterior cervical discectomy and fusion; TDR: total disc replacement.

Variable	Number of patients (n)	Percentage (%)
Total number of patients	20	100
Double-level surgery	17	85
Three-level surgery	3	15
Surgery performed at non-contiguous levels	3	15
ACDF performed cranial to TDR	8	40
ACDF performed caudal to TDR	12	60
Most common combination: C4-C5 TDR + C5-C6 ACDF	6	30
Second most common combination: C5-C6 ACDF + C6-C7 TDR	5	25
Mean duration of surgery (minutes)	125.8	Standard deviation = ± 20.5
Mean intra-operative blood loss (mL)	138	Standard deviation = ± 62.6

All the patients had improvement in terms of clinical parameters at the follow-up in terms of the mean VAS, NDI, and mJOA scores, as shown in Table 3.

**Table 3:** Clinical outcome scores. The table shows the mean (± standard deviation) Visual Analog Scale (VAS) for pain, Neck Disability Index (NDI), and modified Japanese Orthopaedic Association (mJOA) scores before surgery and at the last follow-up visit, demonstrating significant improvement in pain, disability, and neurological function in all 20 patients. VAS: Visual Analog Scale; NDI: Neck Disability Index; mJOA: modified Japanese Orthopaedic Association.

Outcome measure (mean ± SD)	Pre‑operative	Last follow‑up
VAS (pain)	6.8 ± 1.3	2.4 ± 0.7
NDI (disability)	38 ± 5.7	12.6 ± 2.8
mJOA (neurological function)	10.6 ± 2.4	14.4 ± 1.6

On radiological evaluation of the patients at the follow-up, the mean C2-C7 ROM was found to be 38.2° ± 9.6°. The mean ROM at the superior adjacent level was 8.2° ± 3.9°, whereas the mean ROM at the inferior adjacent segment was 9.6° + 4.3°. The radiological parameters at follow-up are summarised in Table 4.

**Table 4:** Radiological parameters at follow up. The table presents the mean (± standard deviation) sagittal range of motion (ROM) at C2-C7 overall, at the superior adjacent level, and at the inferior adjacent segment measured radiologically at the last follow‑up visit. ROM: range of motion.

Radiological parameter	Mean ± SD
C2-C7 ROM	38.2° ± 9.6°
ROM at superior adjacent level	8.2° ± 3.9°
ROM at inferior adjacent segment	9.6° ± 4.3°

Six (30%) patients out of the cohort of 20 patients showed some evidence of subsidence of the disc prosthesis, while two (10%) patients exhibited subsidence of the cage, as seen on the lateral cervical radiographs at the follow-up. However, none of these patients had any clinical complaints. The subsidence rate at the follow-up has been summarised in Table 5.

**Table 5:** Subsidence at the follow-up. Radiographic evidence of subsidence was assessed on follow-up lateral cervical radiographs. Values are expressed as the number of patients (n) and percentage (%). Subsidence was defined as the settling of the disc prosthesis or cage into the adjacent vertebral endplates.

Radiographic finding at follow-up	Number of patients (n)	Percentage (%)
Disc prosthesis subsidence	6	30
Cage subsidence	2	10
Patients with subsidence having clinical complaints	0	0

One patient had developed dysphonia immediately postoperatively, while four patients had transient dysphagia, which resolved in a few days. No evidence of heterotopic ossification was found in the follow-up period in all the patients.

## Discussion

The mobile segments of the cervical spine adjacent to the fused segment are associated with high stress to compensate for the overall mobility of the cervical spine. Increasing the number of fusion levels is thought to be associated with increased stress over the remaining mobile segments [11,12,25]. TDR has been shown to reduce the adjacent segment stresses by preserving the mobility at the pathological segment and reducing the re-operation rates [26].

**Figure 1:**
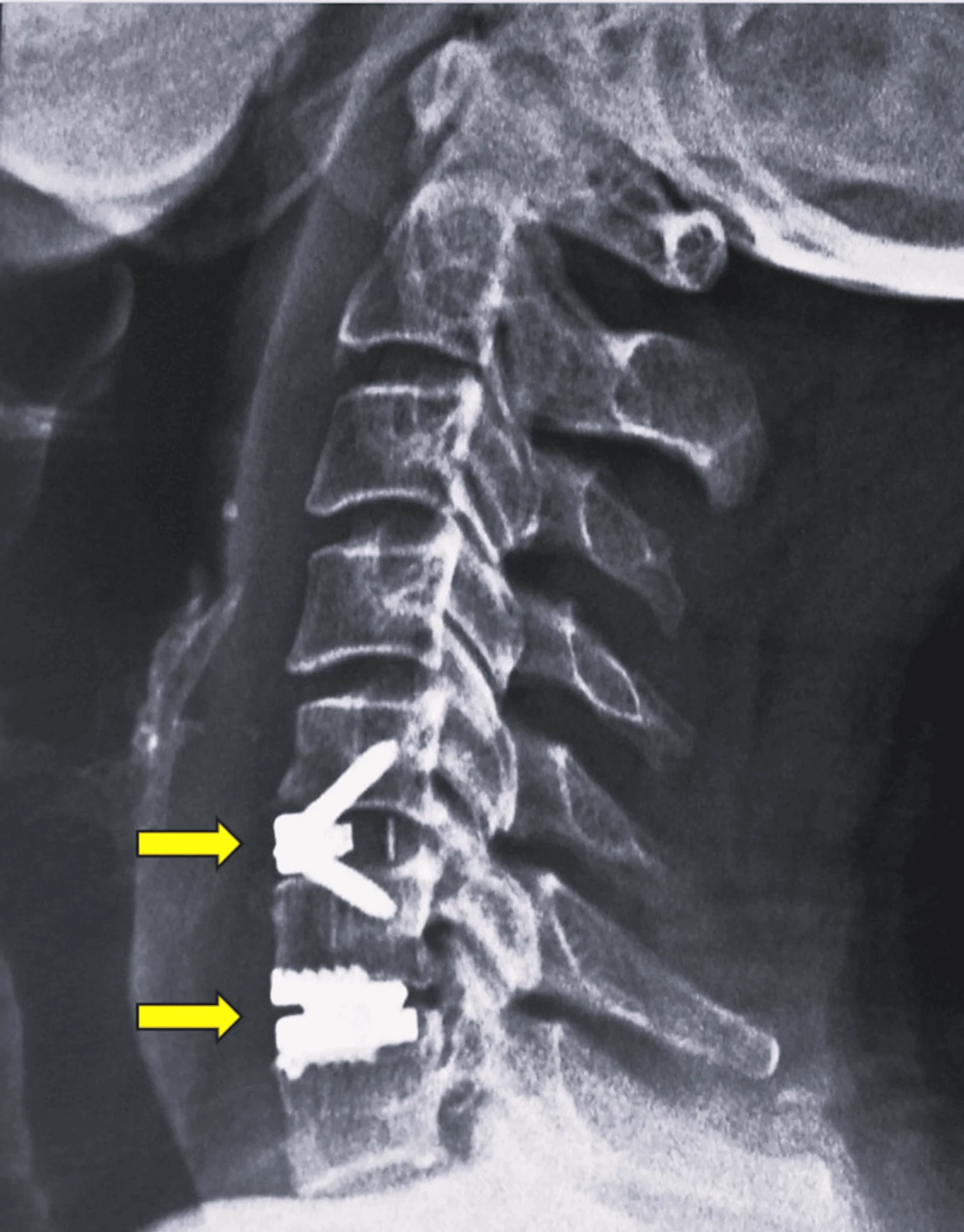
A lateral radiograph of the cervical spine of a patient who underwent hybrid anterior cervical surgery. The upper arrow mark represents the ACDF done at the C5-C6 level, while the lower arrow represents the TDR done at the C6-C7 level. ACDF: anterior cervical discectomy and fusion; TDR: total disc replacement.

Philips et al. found in their prospective multicentric study that TDR adjacent to the prior fused segments is well tolerated, and early outcomes are comparable with those of primary TDR [14]. Although an early in vitro biomechanical study by Martin et al. found that TDR placed adjacent to a two-level fusion is subjected to a more challenging biomechanical environment as compared to the standalone TDR [27]. Lee et al. found in their biomechanical study that the motion response of TDR adjacent to fusion is comparable with that of stand-alone TDR [15]. Later, Hey et al. compared clinical outcomes of hybrid surgery with those of ACDF and TDR in matched patients and found that outcomes of hybrid surgery are comparable to ACDF and TDR in terms of safety and feasibility [21]. Our results of clinical outcomes are consistent with the existing literature. Figure 1 shows a lateral radiograph of the hybrid anterior cervical surgery.

**Figure 2:**
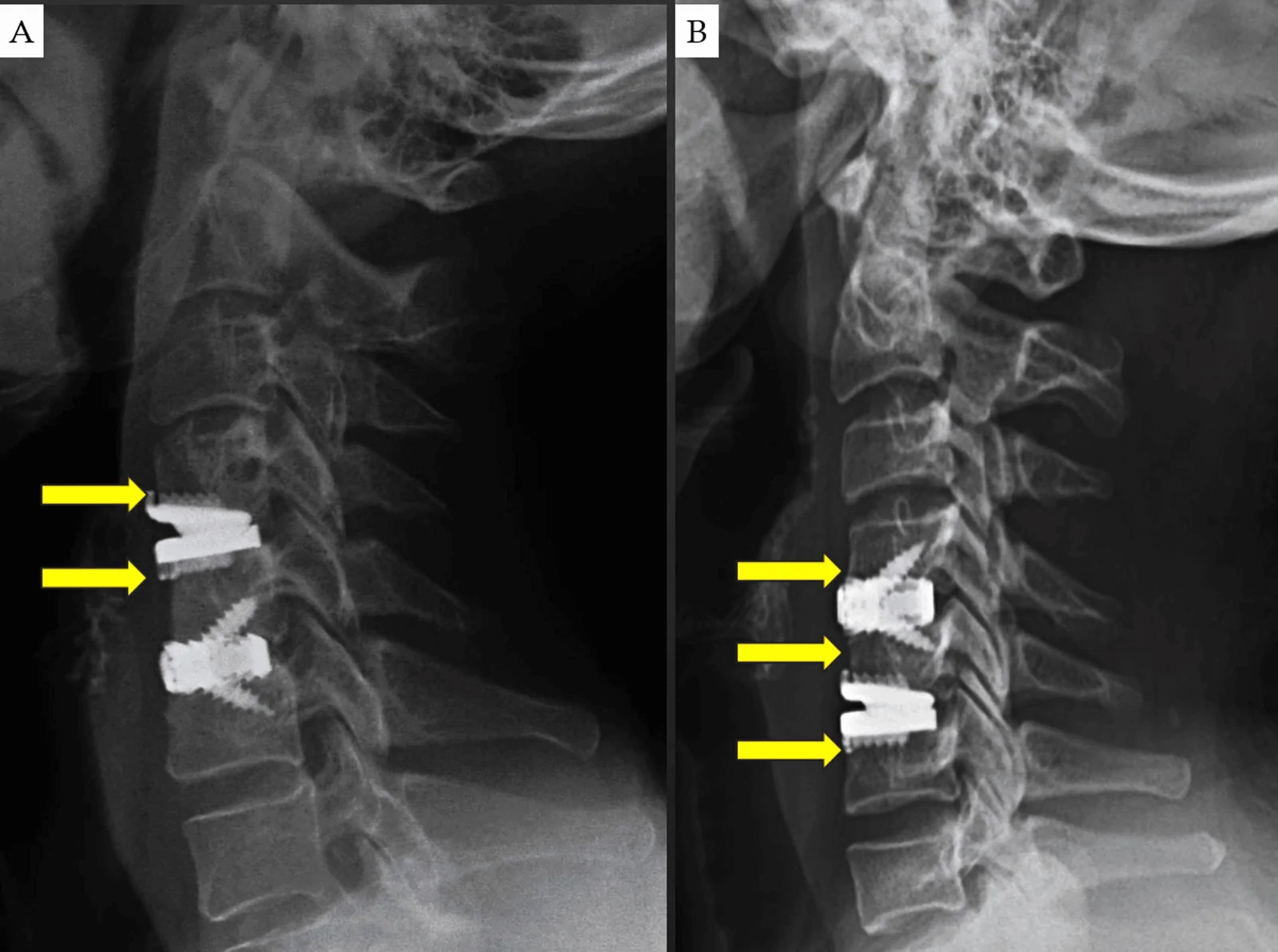
Lateral radiographs of the cervical spine showing subsidence at the time of follow-up. (A) represents subsidence of the disc prosthesis alone, while (B) shows subsidence of the disc prosthesis as well as the cage at the level of fusion. The arrow marks represent subsidence into the adjacent end plates in (A) and (B).

Martin et al. noted higher flexion and extension moment loads adjacent to the disc prosthesis at the fusion level than in standalone TDR. If the disc prosthesis is unable to accommodate such high moment loads, it can lead to impingement of the prosthesis into the end plates and, hence, subsidence [27]. Mende et al. compared hybrid surgery with double-level fusion and found a higher incidence of implant subsidence in the fusion group. They also noted a higher rate of revision surgeries in the hybrid group, which were mostly cage-related [13]. Of the 20 patients in our study, six (30%) showed evidence of disc prosthesis subsidence on cervical spine lateral radiographs, while two (10%) patients had subsidence of the cage without any clinical complaints. We believe this to be consistent with the findings of the biomechanical study of Martin et al. [27]. An example of the same is shown in Figure 2.

The clinical improvements are comparable between the fusion and hybrid surgery groups, as evident by a systematic review and meta-analysis by Lu et al. [28] and supported by various other studies [13,21,29]. Michalopoulos et al. noted that hybrid surgery is non-inferior in comparison to fusion during the early follow-up period in terms of rates of readmission and revision surgeries [4]. Our results show clinical improvement in terms of VAS, NDI, and mJOA scores, comparable to our experience with single-level ACDF and TDR surgeries [30].

**Figure 3:**
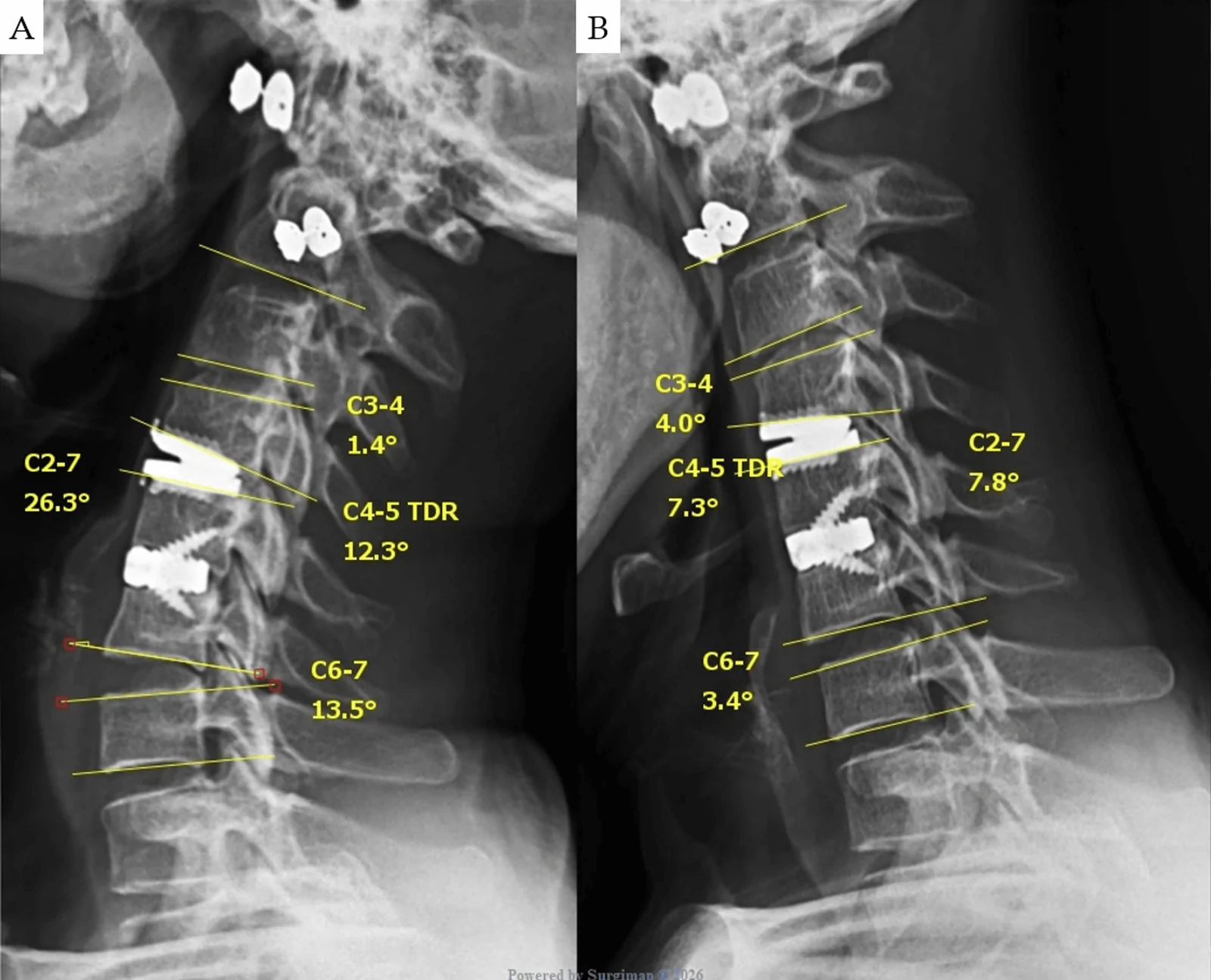
A lateral radiograph of the cervical spine in extension (A) and flexion (B) of a representative case at the follow-up visit three years after surgery, who underwent C4-C5 total disc replacement (TDR) and C5-C6 anterior cervical discectomy and fusion (ACDF). The C2-C7 ROM was 34.1°. The ROM at the superior adjacent segment (C3-C4) was 5.4°, while the same at the inferior adjacent segment (C6-C7) was 16.9°. There is mobility of 5.2° at the TDR level (C4-C5), without any evidence of heterotopic ossification. TDR: total disc replacement; ROM: range of motion.

Since the patients in this study are evaluated at a minimum follow-up of two years, it is unreasonable to assess the rate of ASD, the incidence of which should be practically evaluated at a longer follow-up period of 5-10 years [6]. Therefore, we evaluated the ROM at the superior and inferior adjacent segments to assess any hypermobility that could accelerate spondylosis and lead to ASD, as shown in Figure 3. In the systematic review and meta-analysis by Lu et al., which included eight studies and 169 patients undergoing hybrid surgery, the ROM of the superior and inferior adjacent segments was found to be less in cases that underwent hybrid surgery than in fusion cases [28]. Our evaluation of the ROM at the superior and inferior adjacent segments was found to be comparable to the existing literature.

Xiong et al. found that the hybrid surgery construct could reduce the motion compensation of the adjacent segments and could maintain the global ROM of the cervical spine effectively [28]. From their meta-analysis, Lu et al. showed that the C2-C7 ROM was greater after hybrid surgery as compared to fusion [28]. In this study, the mean C2-C7 ROM was 38.2° ± 9.6°.

The clinical and radiological parameters were compared between the patients who underwent TDR cranial to the ACDF or caudal to it. The same was one of the key findings of the biomechanical study by Lee et al. They did not find a significant difference in the response of the prosthesis when the fused level was cranial or caudal to the TDR [15]. In our practice, for patients with multi-level cervical disc degeneration, ACDF is done at the level with a greater degree of degeneration, while the other level is managed with TDR, whether cranial to the fusion or below it, provided that it is not contraindicated. Secondarily, in cases where contiguous levels are at a similar degree of degeneration, we tend towards preserving mobility by TDR at the segment with more inherent mobility (e.g., C5-C6) and fuse the other level by ACDF.

Our results show that C4-C5 TDR with C5-C6 ACDF was the most common combination, followed by C5-C6 ACDF and C6-C7 TDR. It represents that the middle segment of the cervical spine, having the most pronounced capacity of motion, manifests cervical spondylosis more, especially at the C5-C6 and C6-C7 levels [31]. C5-C6 is usually the apex of the cervical lordosis and is subjected to accelerated degeneration relative to the other levels. Thus, on clinical presentation, in our experience, C5-C6 is found to be affected to a greater degree than C6-C7 or C4-C5 levels [30]. Hence, C5-C6 is the most common level undergoing ACDF among our study patients.

This study has some limitations, including the retrospective nature of the study, a single-centre study, a small sample size, and the lack of a control group. We acknowledge that these limitations may prevent powerful statistical conclusions and limit the generalisability of the results. However, this study represents the objective clinical improvement in terms of VAS, NDI, and mJO scores. The radiological evaluation represents the preservation of the C2-C7 ROM and no evidence of compensatory hyperkinesis at the adjacent segments. Further prospective, multi-centric, long-term studies are warranted with a control group to reiterate the advantages of the hybrid anterior cervical surgery.

## Conclusions

The hybrid anterior cervical surgery for multilevel cervical disc disease, combining the ACDF with TDR, is a safe and effective procedure. It is associated with clinical improvement in terms of VAS, NDI, and mJOA scores, along with preservation of the overall C2-C7 range of motion and avoiding hyperkinesis at the superior and inferior adjacent segments. However, subsidence of the disc prosthesis is noted in the follow-up, probably due to high stress on the implant. Although none of the patients with radiological subsidence had any clinical complaints, long-term follow-up is yet to be seen. On the basis of the clinical and radiological outcomes of our study, hybrid anterior cervical surgery can be a viable option to consider for the management of multilevel cervical disc disease.
